# Nocturnal hypoxia and the success rate of standard atrial fibrillation treatment: a case report

**DOI:** 10.1186/s13256-015-0616-6

**Published:** 2015-06-06

**Authors:** Bülent Güçyetmez, Hakan Korkut Atalan, Hikmet Aloglu, Adnan Kelebek, Tayfun Açıl

**Affiliations:** 1 Department of Anesthesiology, Acıbadem University Faculty of Medicine, Istanbul, Turkey; 2 Intensive Care Unit, Acibadem International Hospital, Istanbul Cad No. 82 Yesilkoy, 34149 Istanbul, Turkey; 3 Intensive Care Unit, Ataşehir Memorial Hospital, Vedat Gunyol Cad No. 28 Kucukbakkalkoy Atasehir, 34758 Istanbul, Turkey; 4 Department of Neurology, Medicalpark Bahçelievler Hospital, Kültür Sok No. 1 Bahçelievler, 34160 Istanbul, Turkey; 5 Department of Cardiology, Acibadem International Hospital, Istanbul Cad No. 82 Yesilkoy, 34149 Istanbul, Turkey

**Keywords:** Atrial fibrillation, Nocturnal hypoxia, Sleep apnea-hypopnea syndrome

## Abstract

**Introduction:**

Sleep apnea-hypopnea syndrome (SAHS) is one of the extracardiac reasons of atrial fibrillation (AF), and the prevalence of AF is high in SAHS-diagnosed patients. Nocturnal hypoxemia is associated with AF, pulmonary hypertension, and nocturnal death. The rate of AF recurrence is high in untreated SAHS-diagnosed patients after cardioversion (CV). In this study, we present a patient whose SAHS was diagnosed with an apnea test performed in the intensive care unit (ICU) and who did not develop recurrent AF after the administration of standard AF treatment and bi-level positive airway pressure (BiPAP).

**Case presentation:**

A 57-year-old male hypertensive Caucasian patient who was on medical treatment for 1.5 months for non-organic AF was admitted to the ICU because of high-ventricular response AF (170 per minute), and sinus rhythm was maintained during the CV that was performed two times every second day. The results of the apnea test performed in the ICU on the same night after the second CV were as follows: apnea-hypopnea index (AHI) of 71 per hour, minimum peripheral oxygen saturation (SpO_2_) of 67%, and desaturation period (SpO_2_ of less than 90%) of 28 minutes. The patient was discharged with medical treatment and nocturnal BiPAP treatment. The results of the apnea test performed under BiPAP on the sixth month were as follows: AHI of 1 per hour, desaturation period of 1 minute, and minimum SpO_2_ of 87%. No recurrent AF developed in the patient, and his medical treatment was reduced within 6 months. After gastric bypass surgery on the 12th month, nocturnal hypoxia and AF did not re-occur. Thus, BiPAP and medical treatments were ended.

**Conclusions:**

SAHS can be diagnosed by performing an apnea test in the ICU. SAHS should be investigated in patients developing recurrent AF after CV. Recovery of nocturnal hypoxia may increase the success rate of standard AF treatment.

## Introduction

Atrial fibrillation (AF) is treated with rhythm and speed control, thromboembolic protection, direct current, and pharmacological cardioversion (CV). Sleep apnea-hypopnea syndrome (SAHS) is one of the extracardiac reasons for AF [1]. AF prevalence is known to be high in SAHS-diagnosed patients [2, 3]. Nocturnal hypoxemia is associated with AF, pulmonary hypertension, and nocturnal death [4-6]. AF recurrence has been reported to be high (82%) in untreated SAHS-diagnosed patients after CV [7]. In our case, the patient was admitted to the intensive care unit (ICU) because of high-ventricular response AF. After CV, SAHS was diagnosed and no recurrent AF was detected after nocturnal bi-level positive airway pressure (BiPAP) treatment administered together with medical treatment.

## Case presentation

**Fig. 1:**
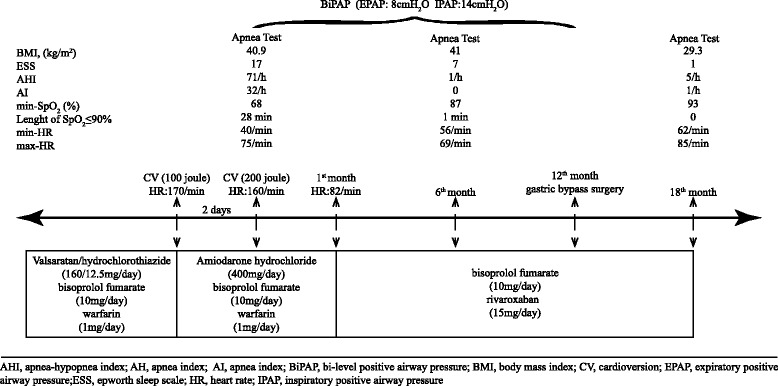
The effects of sleep apnea-hypopnea syndrome treatment on atrial fibrillation treatment. AH, apnea index; AHI, apnea-hypopnea index; AI, apnea index; BiPAP, bi-level positive airway pressure; BMI, body mass index; CV, cardioversion; EPAP, expiratory positive airway pressure; ESS, Epworth sleepiness scale; HR, heart rate; IPAP, inspiratory positive airway pressure; SpO_2_, peripheral oxygen saturation

A 57-year-old male hypertensive Caucasian patient on medical treatment for 1.5 months for non-organic-related AF was hospitalized in the ICU because of high-ventricular response AF (170 per minute). Biphasic CV with 100J was performed on the patient under medical treatment, and sinus rhythm was maintained. The patient had high-ventricular response AF (160 per minute) again after 2 days, biphasic CV with 200J was conducted again, and sinus rhythm was maintained. An apnea test (AT) was performed on the same night because frequent apneas were observed during sedation, and his body mass index (BMI) was 40.9kg/m^2^. The results were as follows: apnea-hypopnea index (AHI) of 71 per hour, desaturation period of 28 minutes, and minimum peripheral oxygen saturation (SpO_2_) of 68 %. The patient was discharged with nocturnal BiPAP—expiratory positive airway pressure of 8cm H_2_O and inspiratory positive airway pressure of 14cm H_2_O—and medical treatment. The patient was in sinus rhythm at the 1-month follow-up; thus, medication doses were decreased, AT was performed under BiPAP at the 6-month follow-up, and the results were as follows: AHI of 1 per hour, desaturation period of 1 minute, and minimum SpO_2_ of 87 %. The patient did not have AF within these 6 months. At the 12th month, the patient decided to undergo gastric bypass surgery. Up to the time of surgery, nocturnal BiPAP and medical treatment were continued. Within 6 months after the medical surgery, the patient lost 40kg and only medical treatment was continued. In the AT conducted in the 18th month (via spontaneous breathing), results in the range of AHI of 5 per hour, apnea index of 1 per hour, and minimum SpO_2_ of 93 % were observed; hence, bisoprolol fumarate was ended (Fig. 1).

## Discussion

Age, obesity, male gender, hypertension, coronary disease, and congestive heart failure are the common risk factors for both AF and SAHS [8]. And nocturnal hypoxia is known to increase the rate of AF prevalence [2, 3, 6, 7]. Recurrent AF developed despite the medical treatment and CV in the patient who had a risk of AF because of obesity and hypertension. Thus, the patient underwent an AT in the ICU in order to investigate SAHS. An AT can easily be applied under ICU conditions and is an affordable method through which AHI, less-than-90 % desaturation period, minimum SpO_2_, and minimum and maximum heart rate can be recorded [9]. Severe SAHS and nocturnal hypoxia were diagnosed in the patient, who underwent an AT on the same night after the second CV. Thus, the administration of nocturnal BiPAP treatment was decided in addition to the standard AF treatment. Kanagala *et al*. showed that the rate of AF recurrence was 82 % 1 year after CV in the untreated SAHS-diagnosed patients but regressed to 42 % after the treatment of SAHS [7]. Besides, in the present study, AF recurrence was associated with the desaturation period that was below 90 %. In the present study, it is remarkable that no AF recurrence developed during the 6 months through which nocturnal BiPAP treatment was administered and the medical treatment was reduced. In the AT performed under nocturnal BiPAP treatment at the sixth month, an improvement was observed in AHI, minimum SpO_2_, and less-than-90 % desaturation period. After the nocturnal BiPAP treatment, not only did the patient develop no recurrent AF but also medical treatment could be reduced and the patient’s quality of life could be increased as the Epworth sleepiness scale regressed from 17 to 7. Besides, we can say that decreased BMI could be the main reason for increased quality of life because BiPAP and medical treatment could be ended after the gastric bypass surgery.

Apnea is known to cause a sudden decline in the intrathoracic pressure, an increase in the left ventricular transmural pressure, vasoconstriction, and sympathetic hyperactivity due to arousal [10]. As a result, diastolic dysfunction, intrathoracic pressure changes, severe hypoxemia, and increased adrenergic state cause fibrosis, distention, structural, and electrical atrial remodeling formation [11-13]. The treatment of sleep apnea prevents intrathoracic pressure fluctuations and autonomic dysfunction and helps to recover atrial myocardium, diastolic ventricular functions, pro-inflammatory state, and oxidative stress [10, 11, 13-15].

We are of the opinion that the relationship between the nocturnal hypoxia and AF should be evaluated through tissue oxygen delivery (DO_2_). The results acquired by Kanagala *et al*. indicate the importance of DO_2_ in the nocturnal hypoxia-AF relationship. DO_2_ is calculated by multiplying the cardiac index (CI) by arterial oxygen content (CaO_2_) [16]. Myocardial tissue uses NADH for the production of mitochondrial energy [17]. Hypoxia causes a decrease in DO_2_, which enables oxidative phosphorylation to endure and results in blocking the electron transport chain and NADH production [18]. Although a recovery is obtained in CI with the standard treatment of AF, untreated nocturnal hypoxia indicates persistence in an insufficient DO2 state in the myocardium cell. This might explain recurrent AF despite standard treatment. The recovery of nocturnal hypoxia together with standard AF treatment may increase the efficiency of AF treatment by improving CaO_2_ and consequently DO_2._ It may even help to reduce the medical treatment as in our case. Thus, the investigation of nocturnal hypoxia together with standard treatment of AF and simultaneous treatment may increase the success rate of the treatment.

## Conclusions

AF is a clinical status that is life-threatening and hence its treatment is obligatory. Administration of AF treatment without treating nocturnal hypoxia would reduce the success of the treatment. Nocturnal BiPAP treatment may increase the success of AF treatment by reducing nocturnal hypoxia and DO_2_. Besides, increased BMI can be the main reason for SAHS, nocturnal hypoxia, and AF. That is why, before gastric surgery, nocturnal BiPAP treatment can also be used for safe preoperative preparation.

## Consent

Written informed consent was obtained from the patient for publication of this case report. A copy of the written consent is available for review by the Editor-in-Chief of this journal.

## Abbreviations

AF: atrial fibrillation
AHI: apnea-hypopnea index
AT: apnea test
BiPAP: bi-level positive airway pressure
BMI: body mass index
CaO_2_: arterial oxygen content
CI: cardiac index
CV: cardioversion
DO_2_: oxygen delivery
ICU: intensive care unit
SAHS: sleep apnea-hypopnea syndrome
SpO_2_: peripheral oxygen saturation
